# Population Pharmacokinetics of Sildenafil in Dogs With Naturally Occurring Pulmonary Hypertension

**DOI:** 10.1111/jvp.70057

**Published:** 2026-02-24

**Authors:** Mariko Yata, Teresa C. DeFrancesco, John D. Bonagura, Mark G. Papich

**Affiliations:** ^1^ Department of Clinical Sciences North Carolina State University, College of Veterinary Medicine Raleigh North Carolina USA; ^2^ Department of Molecular Biomedical Sciences North Carolina State University, College of Veterinary Medicine Raleigh North Carolina USA

**Keywords:** canine, dog, pharmacokinetics, pulmonary hypertension, sildenafil

## Abstract

The pharmacokinetics of sildenafil are ill‐defined in dogs with naturally occurring pulmonary hypertension (PH). Because the plasma concentrations of sildenafil have not been reported for dogs with this disease, this study aimed to describe the population pharmacokinetics of sildenafil in a sample of dogs with PH. Twenty client‐owned dogs with spontaneous PH associated with diverse comorbidities and receiving orally administered sildenafil were enrolled in a prospective, open‐label, steady‐state population pharmacokinetic study. Dogs underwent a sparse‐sampling blood collection protocol after the morning dose of sildenafil. Plasma sildenafil concentrations were determined using high‐pressure liquid chromatography and mass spectrometry. Population pharmacokinetic analysis with nonlinear mixed effects modeling was performed, and selected covariates were evaluated in the model. Sildenafil was rapidly absorbed (absorption half‐life 1.1 h) but variable (CV 94%) and reached maximal plasma concentrations at 2.51 h. The estimated elimination half‐life was 2.9 h. However, substantial individual variability in pharmacokinetics of sildenafil was evident, unexplained by the covariates examined, and attributed primarily to the high variability in absorption. This finding supports the need for a large‐scale study to identify the source of this variability to better guide therapy in poor responders to initial therapy with this drug.

## 1 Introduction

Pulmonary hypertension (PH) describes the presence of abnormally elevated pressures within the pulmonary vasculature and arises chronically as a sequela of cardiac, lung, and pulmonary vascular diseases. Specific causes of PH have been categorized into six major groups by a veterinary consensus statement (Reinero et al. 2020) with suggested treatments for each category. Sildenafil is a phosphodiesterase type‐V inhibitor that induces selective pulmonary vasodilation by increasing intracellular cyclic guanosine monophosphate. Presently, sildenafil is the most prescribed and reported pulmonary arterial vasodilator treatment for dogs with clinical signs related to PH. This drug has been recommended in the treatment of symptomatic PH caused by pulmonary arterial hypertension (Group I), hypoxia or various respiratory disorders (Group III), and potentially in PH caused by embolic, parasitic, and multifactorial diseases (Groups III to VI). Sildenafil can be considered in some dogs with PH associated with concurrent left‐sided heart disease (Group II) provided active pulmonary edema is not present (Reinero et al. 2020).

The pharmacokinetics of sildenafil have been evaluated in healthy dogs. Sildenafil was rapidly absorbed, reaching a peak in 1–2 h, with moderate oral bioavailability (~50%) owing to extensive first‐pass metabolism following oral dosing (Akabane et al. 2018; Al‐Mohizea et al. 2015; Walker et al. 1999; Yang et al. 2018). Bioavailability was lower when administered with food (Akabane et al. 2018). There was a large volume of distribution of approximately 5 L/Kg and an elimination half‐life (*T*_1/2_) of approximately 3–5 h (Akabane et al. 2018; Walker et al. 1999; Yang et al. 2018). Maximal plasma concentrations (*C*_max_) and area under the curve of sildenafil (AUC_inf_) were higher than predicted when larger doses were administered, suggesting pharmacokinetic nonproportionality between 1 and 4 mg/kg. This was attributed to saturation of hepatic metabolism, but unconfirmed (Akabane et al. 2018). Typical dosages administered clinically to dogs range from 0.5 to 3 mg/kg by mouth every 8–24 h (Bach et al. 2006).

Despite widespread use in clinical practice, there are no published reports on the pharmacokinetics of sildenafil in dogs with naturally occurring PH. In the setting of clinically relevant PH, hepatic and gastrointestinal congestion and reduced cardiac output might alter sildenafil absorption and hepatic clearance. A study evaluating sildenafil pharmacokinetics in a canine model of chronic embolic PH described proportional increase in *C*_max_ and AUC_inf_ between 1 and 4 mg/kg (Akabane et al. 2020), which contrasts with what was described earlier in healthy dogs (Akabane et al. 2018). This abolishment of non‐proportionality was presumably due to reduced absorption of the drug, resulting in failure to reach saturation of hepatic metabolism. Additionally, the impact of concurrent medications and comorbidities on the pharmacokinetics of sildenafil in dogs is also unknown. Further research into the pharmacokinetics of sildenafil in dogs with PH could elucidate causes of between‐subject (inter‐individual) variation in pharmacokinetics to better inform the dosing recommendations for this drug. Accordingly, we undertook this study to describe the population pharmacokinetics of sildenafil in dogs with naturally occurring pulmonary hypertension. Because these were client‐owned animals with clinical cardiovascular disease, it was not possible to sample intensely as would be typical of a pharmacokinetic study in healthy research dogs. Therefore, a sparse sampling approach was used with animals sampled at various time points to capture the concentrations across an 8‐h interval (Roy and Ette 2005). We selected the NLME analysis approach because it can accommodate sparse sampling and unbalanced sample points per dog (Owen and Fiedler‐Kelly 2014).

## 2 Materials and Methods

### 2.1 Study Design

This study was a prospective, open‐label, steady‐state population pharmacokinetic study of sildenafil in dogs undergoing chronic therapy of moderate to severe PH. It was conducted at the North Carolina State University Veterinary Hospital between January 2021 and January 2022.

### 2.2 Animals

Twenty client‐owned dogs with previously diagnosed PH that had been treated with sildenafil for a minimum of 3 days were enrolled in this study. All dogs were historically diagnosed with intermediate to high clinical and echocardiographic probability of PH based on the 2020 American College of Veterinary Internal Medicine guidelines on the diagnosis of PH (Reinero et al. 2020) by board‐certified veterinary cardiologists or cardiology residents under direct supervision. This study was approved by the North Carolina State University Animal Ethics Committee (IACUC # 20‐430), and informed owner consent was obtained for each dog prior to enrollment.

Exclusion criteria for the study included administration of drugs that may affect gastrointestinal absorption such as H_2_‐receptor blockers, sucralfate, and proton pump inhibitors within 1 week of the study; administration of drugs that can affect hepatic metabolism such as phenobarbital, rifampin, and ketoconazole; unstable disease requiring escalation or addition of any medications within 1 week of the study; aggressive dogs or those in which venipuncture and echocardiography were not readily tolerated; dogs weighing < 4 kg; dogs with pre‐existing gastrointestinal disease such as inflammatory bowel disease; current gastrointestinal signs such as vomiting and diarrhea; unstable pets in which research interventions should be avoided, such as those with actively decompensated left‐sided congestive heart failure (CHF) or those with moderate to severe respiratory distress.

### 2.3 Schedule of Events

Prior to the day of the study, dogs were treated with sildenafil (Sildenafil 20 mg tablets, Amneal Pharmaceuticals, Bridgewater, NJ) at 0.8–3.6 mg/kg orally every 8 h for a minimum of 3 days. The dose of sildenafil required was dictated by clinical need and chosen by the treating cardiologist prior to enrollment into the study.

Dogs were fasted overnight, and usual morning medications were withheld prior to presentation on the day of the study. However, if required, dogs were permitted to have trazodone (range 1.6–5.1 mg/kg) administered at home to minimize stress of hospitalization prior to presentation. Following initial physical examination, dogs underwent an echocardiographic exam, and a blood sample was taken for packed cell volume and a complete biochemical panel, which was performed at the North Carolina State University clinical pathology laboratory. A pre‐treatment blood sample (time 0) was taken in those pre‐determined to have this sample drawn. The morning dose of sildenafil was then administered, and each dog underwent two to three further venipunctures for blood collection at designated times (either at 20, 40 min, 1, 1.5, 2, 3, 4, 6, or 8 h post‐dosing) based on pre‐determined randomized allocations (Table S1). Thus, each dog had a total of three blood samples obtained for pharmacokinetic analysis and one for packed cell volume and serum biochemistry analysis. This sparse‐sampling design was used to minimize stress associated with repeated venipuncture on the dogs.

Food and any other usual morning medications were administered 3 h after the sildenafil dose. Following acquisition of the final blood sample, dogs were returned to their owners for ongoing care at home.

### 2.4 Analytical Method for Sildenafil

Blood samples were collected in blood tubes containing lithium heparin, immediately refrigerated, and centrifuged at 5500 RCF for 10 min within 3 h of collection to harvest plasma. Plasma was stored frozen at −80°C for up to 24 months prior to sample analysis.

Plasma samples were analyzed for sildenafil using high‐pressure liquid chromatography (HPLC) paired with mass spectrometry detection (LC–MS) using a validated method developed in the Clinical Pharmacology Laboratory at North Carolina College of Veterinary Medicine. This laboratory uses the International Council for Harmonization of Technical Requirements for Pharmaceuticals for Human Use (ICH 1994) (available at: https://database.ich.org/sites/default/files/ICH_Q2‐R2_Document_Step2_Guideline_2022_0324.pdf) and the guidelines published in Chapter 1225 of the United States Pharmacopeia (USP 2023).

Incurred samples, calibration samples and quality control samples were prepared by pipetting 500 μL of plasma into a clean tube and adding 1 mL of acetonitrile. After vortexing, the samples were centrifuged for 5 min, and 1 mL of supernatant was harvested. The supernatant was dried in an evaporator under an air flow of 20 psi at 40°C for 20 min. The dry residue was reconstituted with 250 μL of a mixture of 60/40 (Water/Acetonitrile) and loaded into a Whatman syringeless vial (Whatman UniPrep syringeless filters. Pore size 0.2 μm, Cytiva, USA) for injection. The recovery from the extraction of sildenafil from plasma was approximately 100%.

Following extraction, the samples were injected into the LC–MS system that consisted of a quaternary solvent delivery system at a flow rate of 1.0 mL/min (Agilent 1260 Solvent Delivery System, Agilent Technologies, Wilmington, DE), an autosampler (Agilent 1260 Autosampler, Agilent Technologies, Wilmington, DE), and a mass spectrometer (Agilent 61060A LC/MSD iQ, Agilent Technologies, Wilmington, DE). The mass spectrometer was run in Auto Acquire single ion monitoring mode (SIM) with positive ionization and a mass/charge (m/z) of 475.3. Chromatograms were integrated with a computer program (Agilent OpenLAB CDS software, Agilent Technologies, Wilmington, DE). The column was a reverse‐phase, 4.6 mm × 15 cm C8 column (Zorbax SB‐C18 4.6 × 15; 5 μm) kept at a constant temperature of 40°C. The mobile phase consisted of 30% acetonitrile and 70% 0.1% formic acid in water. Fresh mobile phase was prepared, filtered (0.45 μm), and degassed for each daily run.

Calibration curve samples and quality control samples were prepared by fortifying blank (control) canine plasma with standard solutions of sildenafil. The standard spiking solutions were prepared by dissolving the sildenafil analytical reference standard in pure methanol and creating additional dilutions with a mixture of 60/40 (water/acetonitrile). Spiking solutions were stable in the refrigerator for 8 weeks. The incurred samples were quantified using a calibration curve that consisted of fortified blank canine plasma with seven calibration standards ranging from 5 to 1000 ng/mL and included a blank (0 ng/mL). The limit of quantitation (LOQ) for the assay was 5 ng/mL, determined by the lowest point on the linear calibration curve that met our criteria for acceptance. Quality control (QC) samples were analyzed to ensure that the assay performed according to our acceptance criteria. We analyzed QC samples at a low concentration (10 ng/mL), 2 medium concentrations (50 and 100 ng/mL), and a high concentration (500 ng/mL) anticipated for our results. The accuracy of the assay was 81%, 103.9%, 103.2%, and 99.1% for the low, 2 medium, and high concentrations, respectively. The precision was 17.3%, 9.2%, 10.8%, and 0.65% for the same concentrations, respectively. These values met our acceptance criteria. The low concentration (10 ng/mL) was slightly out of range but the number of incurred samples at this low concentration was insignificant. All incurred samples measured were above the value of the LOQ. Fresh calibration standards and quality control samples were prepared daily for each run.

### 2.5 Pharmacokinetic Analysis of Sildenafil

The analysis was performed using a population pharmacokinetic approach (Owen and Fiedler‐Kelly 2014) and samples analyzed by nonlinear mixed‐effects modeling (NLME) using Phoenix software (Phoenix, NLME version 8.3, Certara, St. Louis, MO) (Mould and Upton 2012).

A naïve averaged analysis was used to obtain initial estimates (data not shown). These initial estimates were used as input for the population pharmacokinetic analysis. This analysis produces the typical value for the population parameters (fixed effects), estimates for the between‐subject variability (random effects), and allows for exploration of the sources of variation. From these initial estimates, the NLME model was fitted to these data. Compartmental analysis of the data from sildenafil administration to the dog was calculated using a one‐compartment model according to Equation (1):

(1) $$C\left(T\right)=\frac{D\cdot \mathrm{Ka}}{V/F\left(\mathrm{Ka}-\mathrm{Ke}\right)}\times \left[e^{-\mathrm{Ke}\cdot T}-e^{-\mathrm{Ka}\cdot T}\;\right]$$

where *C* is the sildenafil concentration at time *T*, *D* is the dose, *V*/*F* is the apparent volume of distribution, Ke is the elimination rate constant, and Ka is the absorption rate constant. Secondary parameters calculated include the elimination and absorption half‐life (*T*_½_), area‐under‐the‐curve (AUC), peak concentration (*C*_MAX_), and time to peak concentration (*T*_MAX_). Apparent oral clearance (CL/F) and *V*/*F* were calculated and presented in our table, but without an accompanying intravenous dose, these values have little meaning. Plasma drug concentrations were assumed to be at steady state because prior doses were administered before we started our blood collection. Therefore, the model was run using the steady‐state input option in Phoenix.

Various models were tested with different error structures to determine the best fit base model using the minimum value of twice the negative log likelihood (−2LL). The model was run in first order conditional estimation extended least squares (FOCE‐ELS) mode. Final model selection was based on goodness of fit plots, diagnostic plots of residuals, scatter plots of predicted vs. observed values, and statistical significance between models using the minimal objective function value (OFV) (Owen and Fiedler‐Kelly 2014).

Inter‐individual (between‐subject) variability (variance of a parameter among different subjects) was expressed using an exponential error model according to Equation (2):

(2) $$\mathrm{Pi}=\theta \;P\times e^{\mathrm{\eta i}\;P}$$

where *P* is the pharmacokinetic parameter of interest for the individual dog (*i*), *θ P* is *θ* (theta), or the typical value (fixed effect) for the population estimate of the parameter of interest, and *η*_*i*_ *P* is the *η* (eta, random effect) for the inter‐individual (between‐subject) differences of the parameter of interest. The *η* values were assumed to be independent and have a normal distribution with a mean of zero and variance of *ω* ^2^. An additive model described the residual random variability (*ε*) of the data, where ε is the residual intra‐subject (within‐subject) variability with a mean of zero and a variance of *σ* ^2^, according to Equation (3).

(3) $$C_{\mathrm{obs}}=C_{\text{pred}}\times \left(1+\varepsilon \right)$$

where *C*_obs_ is the observed concentration for the individual, and *C*_pred_ is the model predicted concentration plus the error value (*ε*).

### 2.6 Additional Pharmacokinetic Analyses

Because there was substantial variation in pharmacokinetics among the dogs, various covariates (listed below) were included in an exploratory analysis to identify potential factors that might explain these differences. Covariates were examined for their effect on between‐subject variability (*η*). In this analysis, covariates were included in the model in a forward stepwise manner and the improvement (reduction) in the OFV was evaluated for a statistically significant effect. Dependent variables were Ka, Ke, and *V*/*F*. Continuous covariates explored were body weight (kg), age, dose (mg/kg), and serum creatinine, ALKP, and ALT. Binary categorical covariates explored were concurrent diuretic administration and concurrent pimobendan administration. The effect of covariates on η was initially assessed using visual examination of box plots (categorical covariates) or covariate plots (continuous covariates) and the shotgun run option on the Phoenix software. The effects of the covariate on a parameter were evaluated based on changes in the −2LL (equivalent to the objective function value in NONMEM) and decreases considered significant at *p* < 0.01. A backward elimination step was used to assess the significance of the covariate, with increased −2LL considered significant at *p* < 0.001. The predictive accuracy of the final model was tested using the visual predictive check (VPC) feature in Phoenix to compare observed quantiles with predicted quantiles predicted by the model. After examining the VPC plots, we concluded that our model was adequate for these data.

## 3 Results

Twenty dogs were enrolled in the study. The median age was 11 years (range 1–16 years) with roughly even sex distribution (*n* = 9 female spayed; *n* = 2 female intact; *n* = 7 male castrated; *n* = 2 male intact). Breeds enrolled included mixed breed dogs (*n* = 7), West Highland White Terrier (*n* = 2), Pekingese (*n* = 2), Shih Tzu (*n* = 2), and one each of Pembrooke Corgi, American Staffordshire Terrier, Miniature Dachshund, Boxer, Terrier, Miniature Pinscher, and Chihuahua.

Based on a full clinical evaluation, the etiology of pulmonary hypertension was classified (Reinero et al. 2020) as undetermined (Group 6, *n* = 6), heartworm disease (Group 5, *n* = 4), lung or airway disease (Group 3, *n* = 4), congenital heart disease (Groups 1 and 2, *n* = 2; one each due to reversed patent ductus arteriosus and ventricular septal defect), mixed heartworm and lung or airway disease (Groups 3 and 5, *n* = 2), and one dog each with both chronic lung disease and confirmed pulmonary thromboembolism (Groups 3 and 4), and ACVIM stage D myxomatous mitral valve disease (Group 2). A median tricuspid regurgitation pressure gradient of 76.5 mmHg (range 49–120) was determined at the time of PH diagnosis in 18 dogs that had measurable tricuspid regurgitation (Reinero et al. 2020). Following initiation of sildenafil, owners reported either resolution or improvement of clinical signs in all dogs, which had included syncope, coughing, respiratory distress, cyanosis, and right‐sided congestive heart failure. The median duration of treatment with sildenafil prior to the study was 57 days (range 14–829). Sildenafil was administered as monotherapy (*n* = 8) or part of multi‐drug therapy (*n* = 12). The median tricuspid regurgitation pressure gradient at the time of the study (while receiving sildenafil) was 49.4 mmHg (range 19–117) as obtained in 16 dogs with measurable tricuspid regurgitation.

The median daily dose of sildenafil was 5.2 mg/kg (range 2.4–10.7). The median dose of sildenafil given on the day of the study was 1.8 mg/kg (range 0.8–3.6, with a CV of 32%). Concurrent medications included pimobendan (*n* = 7), enalapril (*n* = 4), spironolactone (*n* = 2), furosemide (*n* = 2), torsemide (*n* = 1), prednisolone (*n* = 3), doxycycline (*n* = 2), hydrocodone (*n* = 2), and one each of fluoxetine, taurine, theophylline, levothyroxine, diphenhydramine, cetirizine, tacrolimus eye ointment, grapiprant, atenolol, telmisartan, clopidogrel, and carprofen.

Blood samples for pharmacokinetic analysis were acquired from all dogs. One dog (Dog 5) experienced a delay in blood sampling (30 min instead of scheduled 20 min). Otherwise, blood samples from 4 to 7 dogs were used for pharmacokinetic analysis at each time point. The calculated pharmacokinetic parameters are summarized in Table 1. As anticipated, plasma concentrations of sildenafil were detected at baseline in all dogs sampled at this time point (*n* = 5) because steady‐state conditions were assumed. All concentrations were above our criteria for the lower limit of quantification (LOQ). Sildenafil was rapidly absorbed (*T*_1/2_ 1.1 h) but highly variable with CV for Ka of 94%. The concentrations reached a peak (*C*_MAX_) of 258 ng/mL at 2.5 h (*T*_MAX_). The typical value for the population estimated elimination half‐life (*T*_1/2_) was 2.89 h.

**TABLE 1:** Pharmacokinetics of sildenafil in dogs with pulmonary hypertension (*n* = 20) following oral sildenafil (median 1.7 mg/kg, range 0.8–3.6) using Nonlinear Mixed Effects Modeling (NLME), with one compartment model and first order input.

Parameter (units)	Estimate	Shrinkage	CV%
*θ*Ka (/h)	0.62	0.12	93.68
*θV*/*F* (L/kg)	4.03	0.14	15.48
*θ*Ke (/h)	0.24	0.11	20.67
*T*_MAX_ (h)	2.51		
AUC (ng × h/mL)	1967.04		
*C*_MAX_ (ng/mL)	258.71		
CL/F (L/kg/h)	0.97		
Ka *T*_½_ (h)	1.12		
Ke *T*_½_ (h)	2.89		

*Note:* Values for primary parameters of V, Ka, and Ke are represented by the typical values for the population, also designated by “theta”, *θ*.

Abbreviations: AUC, area‐under‐the‐curve; CL/F, clearance per fraction absorbed; *C*_MAX_, peak concentration; h, hours; Ka, absorption rate, with associated half‐life (*T*_½_); Ke, elimination rate, with associated half‐life (*T*_½_); *T*_MAX_, time to peak concentration; *V*/*F*, volume of distribution per fraction absorbed.

**FIGURE 1:**
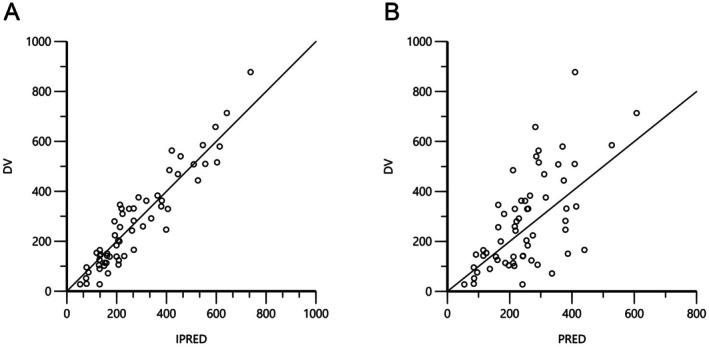
Goodness of fit plots depicting individual (IPRED, panel A) and population (PRED, panel B) predicted values of sildenafil concentrations over time (DV) using the final model derived from the NLME analysis.

**FIGURE 2:**
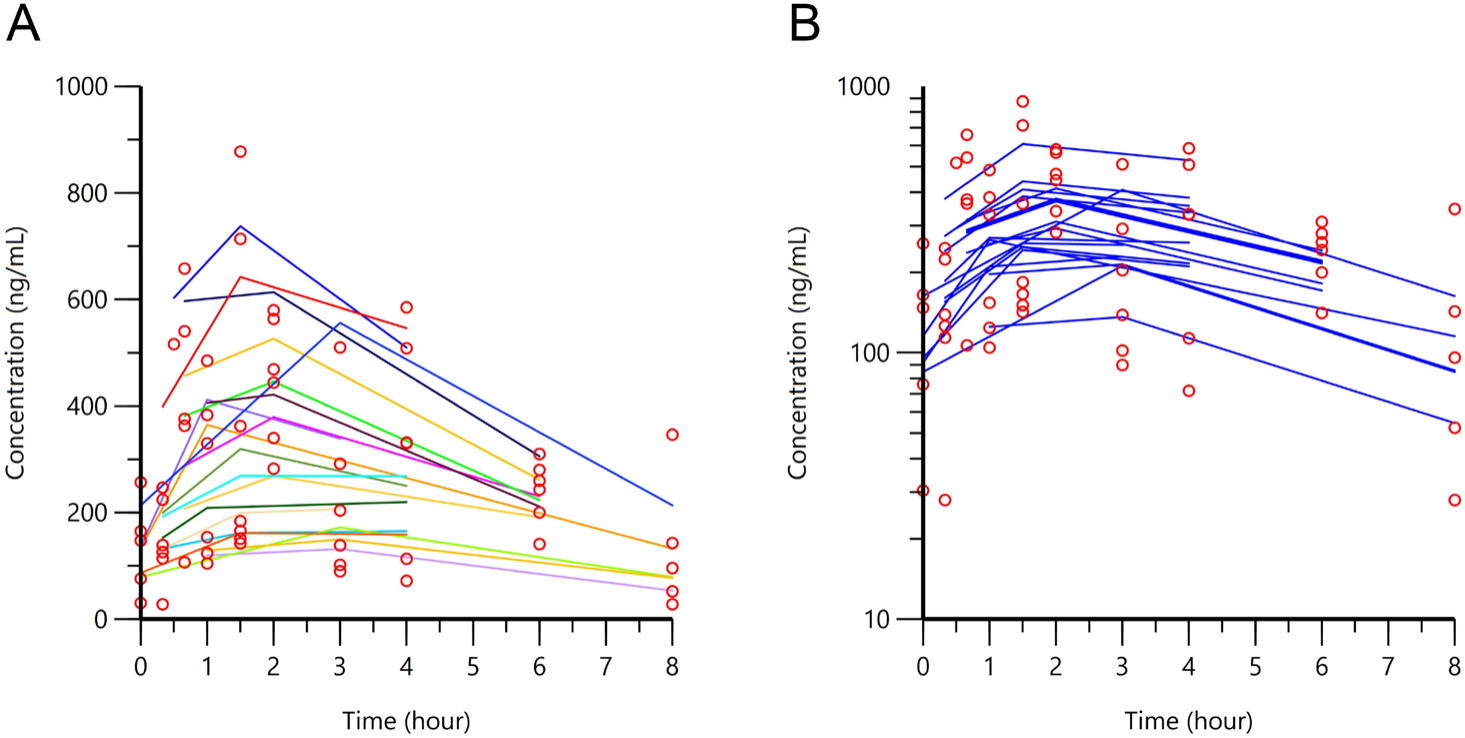
Individual observed versus predicted values of sildenafil following population pharmacokinetic analysis in 20 dogs with pulmonary hypertension dosed with oral sildenafil (median 1.7 mg/kg, range 0.8–3.6). Panel A shows individual predicted values (IPRED), which depict a spaghetti plot of each individual fitted to the model. Open circles represent observed sildenafil concentrations, and lines represent the model‐fitted predictions. Panel B depicts the fitted curve after accounting for between‐subject (random) variability. Note the persistence of unexplained variability (wide spread of data) albeit improvement in the curve fit.

Adequate model fit for the data was assessed via inspection of the goodness of fit plots comparing the individual and population predicted values of sildenafil concentrations over time using the final model derived from the NLME analysis (Figure 1). Analysis also showed acceptable levels of shrinkage (< 20%–30%) (Karlsson and Savic 2007), indicating that we did not over‐parameterize the model. Following population pharmacokinetic analysis, a large degree of between‐subject (inter‐individual) variability was observed, as evidenced by the spread of data on the spaghetti plot showing individual model‐fitted values (Figure 2A). Fitting the curve to account for between‐subject variation improved variability (Figure 2B); however, a large amount of unexplained variability persisted (Figure 2B). Exploration of covariates (body weight, dose, creatinine, ALKP, ALT, age, diuretic administration, pimobendan administration) did not identify any significant factors that could explain this variability using our criteria.

## 4 Discussion

This study evaluated the population pharmacokinetics of oral sildenafil in a clinically diverse group of dogs with naturally occurring PH. Our results showed high individual variability in sildenafil oral pharmacokinetics.

The degree of variability in sildenafil oral pharmacokinetics observed in this study is striking and could contribute towards a variable and inadequate response to the drug in individual dogs. Without additional undue study and accompanying IV studies, we cannot determine the source of the between‐subject variation observed in our results. Our results showed that the highest degree of variability was for the parameter of Ka, the absorption rate (CV 94%), which can be affected by many factors, including feeding, diet, underlying disease, and other medications. The variable timing of our initial samples in the first hour likely also affected this variability. The first blood sample in some dogs was at time zero, but in other dogs, it was either 20 or 40 min after the dose was administered (Table 1). The mg/kg dose varied among dogs with a %CV of approximately 32%, which also may have affected the rate of absorption and likely contributed to the variable concentrations shown in our plots.

Given the known effects of hepatic congestion, reduced cardiac output, and renal disease on bioavailability, metabolism, and clearance of drugs, we hypothesized that these may be significant factors that could explain the degree of variability in the data. We were unable to identify significant factors among the covariates evaluated that could explain the variability using our criteria. However, we acknowledge a major limitation in our study, which is the small number of subjects enrolled (*n* = 20). This may have been an insufficient number to identify significant factors in our sample that affected these pharmacokinetic values (Ogungbenro and Aarons 2008). A larger population of > 50 dogs would have improved the analysis. Furthermore, the heterogeneous sample included dogs with varying etiologies and severity of PH that were receiving different medications and dosages. This cohort does, however, represent the variety of presentations observed in the clinical situation. Samples from this study were stored for up to 24 months prior to analysis, but sildenafil in human plasma remains stable for up to 1 year and through three freeze–thaw cycles (Paul et al. 2005).

Sildenafil has low oral bioavailability in dogs which is subject to first‐pass effects and presumably these factors greatly affected the between‐subject variation observed in the present study. Hepatic clearance can be particularly variable among clinical patients because of changes in hepatic blood flow, the activity of metabolizing enzymes, and the effects of feeding and concurrent medications (Hellriegel et al. 1996). Sildenafil has only ~50%–60% bioavailability in reported literature, and variability in pharmacokinetics has been demonstrated in the spread of data observed in prior studies evaluating sildenafil oral pharmacokinetics in dogs (Akabane et al. 2018, 2020; Yang et al. 2018; Walker et al. 1999). The use of clinical patients in the present study precluded intravenous dosing, and without an accompanying intravenous dose, we could not measure the absolute bioavailability. A more extensive study to evaluate the bioavailability of this drug in a larger group of dogs with spontaneous PH is necessary to identify the impact of various factors on oral pharmacokinetics and inform veterinary clinicians on these effects. Such data might help to predict dogs that are poor responders to therapy with this drug. It should be noted, however, that the presence or absence of the PDE5A:E90K gene polymorphism should first be characterized in dogs enrolled in studies evaluating effect of pharmacokinetics on response to sildenafil because it has been shown that this genetic variation results in reduced response to sildenafil (Ueda et al. 2019; Stern et al. 2014).

Albeit some clinicians prescribing sildenafil as a twice daily drug in practice, administering the drug at dosing intervals of 8 h produced detectable concentrations throughout the 8 h sampling interval of this study. We also showed detectable plasma concentrations of the drug at 0 h. Given previous studies (Akabane et al. 2018, 2020; Walker et al. 1999; Yang et al. 2018) also observed a half‐life of ~3–5 h, a dosing interval of every 8 h would appear to be appropriate for this drug in dogs. More frequent intervals would likely place an additional burden on the owners of these dogs.

## 5 Conclusion

Our dosing schedule of oral sildenafil in a sample of dogs with clinically relevant PH of varying etiologies produced detectable concentrations of the drug throughout the 8 h sampling interval. We observed a high degree of variability in plasma concentrations and oral absorption; however, the cause of this variability is undetermined. In order to identify the cause of this variability, the authors would recommend evaluation of a larger number of dogs (*n* > 50) with spontaneously occurring pulmonary hypertension, more consistent timing of the first sample after administering the dose of sildenafil, more frequent sampling in the absorption phase, limiting the range of mg/kg dose where possible, and considering a narrower range of comorbidities in the inclusion criteria.

## Author Contributions

Mariko Yata: study design, data collection, analysis, writing, and revising the manuscript. Teresa C. DeFrancesco: study design, analysis, writing, and revising the manuscript. John D. Bonagura: study design, analysis, writing, and revising the manuscript. Mark G. Papich: Study design, analysis, writing, and revising the manuscript.

## Funding

This work was supported by the American College of Veterinary Internal Medicine resident research grant.

## Ethics Statement

The authors confirm that the ethical policies of the journal, as noted on the journal's author guidelines page, have been adhered to. The authors confirm that they have adhered to the US standards for the protection of animals used for scientific purposes. This study was approved by the North Carolina State University Animal Ethics Committee (IACUC # 20‐430).

## Conflicts of Interest

The authors declare no conflicts of interest.

## Supporting information


**Table S1:** Pre‐determined randomized allocations for blood sampling for population pharmacokinetic analysis of sildenafil in dogs (*n* = 20) with naturally occurring pulmonary hypertension following an oral dose of sildenafil.

## Data Availability

The data that support the findings of this study are available from the corresponding author upon reasonable request.

## References

[jvp70057-bib-0001] Akabane, R. , T. Sato , A. Sakatani , Y. Miyagawa , H. Tazaki , and N. Takemura . 2018. “Pharmacokinetics of Single‐Dose Sildenafil Administered Orally in Clinically Healthy Dogs: Effect of Feeding and Dose Proportionality.” Journal of Veterinary Pharmacology and Therapeutics 41, no. 3: 457–462. 10.1111/jvp.12487. 29352474

[jvp70057-bib-0002] Akabane, R. , T. Sato , A. Sakatani , et al. 2020. “Pharmacokinetics of Single Dose Sildenafil Orally Administered in Canine Models of Chronic Embolic Pulmonary Hypertension.” Journal of Veterinary Medical Science 82, no. 4: 446–451. 10.1292/jvms.19-0595. PMC7192714 32101822

[jvp70057-bib-0003] Al‐Mohizea, A. M. , A. Ahad , G. M. El‐Maghraby , F. I. Al‐Jenoobi , K. M. AlKharfy , and S. A. Al‐Suwayeh . 2015. “Effects of *Nigella sativa*, Lepidium Sativum and *Trigonella foenum‐graecum* on Sildenafil Disposition in Beagle Dogs.” European Journal of Drug Metabolism and Pharmacokinetics 40, no. 2: 219–224. 10.1007/s13318-014-0199-4. 24719213

[jvp70057-bib-0004] Bach, J. F. , E. A. Rozanski , J. MacGregor , J. M. Betkowski , and J. E. Rush . 2006. “Retrospective Evaluation of Sildenafil Citrate as a Therapy for Pulmonary Hypertension in Dogs.” Journal of Veterinary Internal Medicine 20, no. 5: 1132–1135. 10.1111/j.1939-1676.2006.tb00711.x. 17063705

[jvp70057-bib-0005] Hellriegel, E. T. , T. D. Bjornsson , and W. W. Hauck . 1996. “Interpatient Variability in Bioavailability Is Related to the Extent of Absorption: Implications for Bioavailability and Bioequivalence Studies.” Clinical Pharmacology & Therapeutics 60, no. 6: 601–607. 10.1016/S0009-9236(96)90208-8. 8988062

[jvp70057-bib-0006] ICH . 1994. “International Conference on Harmonisation (ICH) Harmonised Tripartite Guideline: Validation of Analytical Procedures: Text and Methodology.” https://database.ich.org/sites/default/files/Q2%28R1%29%20Guideline.pdf.

[jvp70057-bib-0007] Karlsson, M. O. , and R. M. Savic . 2007. “Diagnosing Model Diagnostics.” Clinical Pharmacology & Therapeutics 82, no. 1: 17–20. 10.1038/sj.clpt.6100241. 17571070

[jvp70057-bib-0008] Mould, D. , and R. Upton . 2012. “Basic Concepts in Population Modeling, Simulation, and Model‐Based Drug Development.” CPT: Pharmacometrics & Systems Pharmacology 1, no. 9: 6. 10.1038/psp.2012.4. PMC3606044 23835886

[jvp70057-bib-0009] Ogungbenro, K. , and L. Aarons . 2008. “How Many Subjects Are Necessary for Population Pharmacokinetic Experiments? Confidence Interval Approach.” European Journal of Clinical Pharmacology 64, no. 7: 705–713. 10.1007/s00228-008-0493-7. 18483725

[jvp70057-bib-0010] Owen, J. S. , and J. Fiedler‐Kelly . 2014. Introduction to Population Pharmacokinetic/Pharmacodynamic Analysis With Nonlinear Mixed Effects Models. John Wiley & Sons, Inc. 10.1038/psp.2014.51 PMC5684957 25531560

[jvp70057-bib-0011] Paul, G. A. , J. S. R. Gibbs , A. R. Boobis , A. Abbas , and M. R. Wilkins . 2005. “Bosentan Decreases the Plasma Concentration of Sildenafil When Coprescribed in Pulmonary Hypertension.” British Journal of Clinical Pharmacology 60, no. 1: 107–112. 10.1111/j.1365-2125.2005.02383.x. PMC1884910 15963102

[jvp70057-bib-0012] Reinero, C. , L. C. Visser , H. B. Kellihan , et al. 2020. “ACVIM Consensus Statement Guidelines for the Diagnosis, Classification, Treatment, and Monitoring of Pulmonary Hypertension in Dogs.” Journal of Veterinary Internal Medicine 34: 549–573. 10.1111/jvim.15725. PMC7097566 32065428

[jvp70057-bib-0013] Roy, A. , and E. I. Ette . 2005. “A Pragmatic Approach to the Design of Population Pharmacokinetic Studies.” AAPS Journal 7, no. 2: 41. 10.1208/aapsj070241. PMC2750978 16353920

[jvp70057-bib-0014] Stern, J. A. , Y. Reina‐Doreste , L. Chdid , and K. M. Meurs . 2014. “Identification of PDE5A:E90K: A Polymorphism in the Canine Phosphodiesterase 5A Gene Affecting Basal cGMP Concentrations of Healthy Dogs.” Journal of Veterinary Internal Medicine 28, no. 1: 78–83. 10.1111/jvim.12256. PMC4895552 24341639

[jvp70057-bib-0015] Ueda, Y. , L. R. Johnson , E. S. Ontiveros , L. C. Visser , C. T. Gunther‐Harrington , and J. A. Stern . 2019. “Effect of a Phosphodiesterase‐5A (PDE5A) Gene Polymorphism on Response to Sildenafil Therapy in Canine Pulmonary Hypertension.” Scientific Reports 9, no. 1: 6899. 31053768 10.1038/s41598-019-43318-z PMC6499771

[jvp70057-bib-0016] USP . 2023. “United States Pharmacopeia. General Chapter, (1225) Validation of Compendial Procedures.” 10.31003/USPNF_M99945_04_01.

[jvp70057-bib-0017] Walker, D. K. , M. J. Ackland , G. C. James , et al. 1999. “Pharmacokinetics and Metabolism of Sildenafil in Mouse, Rat, Rabbit, Dog and Man.” Xenobiotica 29, no. 3: 297–310. 10.1080/004982599238687. 10219969

[jvp70057-bib-0018] Yang, H.‐J. , Y.‐I. Oh , J.‐W. Jeong , K.‐H. Song , T.‐S. Koo , and K.‐W. Seo . 2018. “Comparative Single‐Dose Pharmacokinetics of Sildenafil After Oral and Rectal Administration in Healthy Beagle Dogs.” BMC Veterinary Research 14, no. 1: 291. 10.1186/s12917-018-1617-7. PMC6154896 30249242

