# Supplementary material for: Population Pharmacokinetics of Sildenafil in Dogs With Naturally Occurring Pulmonary Hypertension
Source: J Vet Pharmacol Ther. 2026 Feb 24;49(3):267–73. doi: 10.1111/jvp.70057 (PMC13159767; doi:10.1111/jvp.70057)
Supplement: Supplementary file 1 — Table S1: Pre‐determined randomized allocations for blood sampling for population pharmacokinetic analysis of sildenafil in dogs (n = 20) with naturally occurring pulmonary hypertension following an oral dose of sildenafil. [file JVP-49-267-s001.docx]

Supplementary Table 1: Pre-determined randomized allocations for blood sampling for population pharmacokinetic analysis of sildenafil in dogs (n = 20) with naturally occurring pulmonary hypertension following an oral dose of sildenafil.

| **Dog** | **0 min** | **20 min** | **40 min** | **1 h** | **1.5 h** | **2 h** | **3 h** | **4 h** | **6 h** | **8 h** |
| --- | --- | --- | --- | --- | --- | --- | --- | --- | --- | --- |
| 1 | X |  |  | X |  |  | X |  |  |  |
| 2 |  |  |  | X |  |  | X |  |  | X |
| 3 |  | X |  |  | X |  |  | X |  |  |
| 4 |  |  | X |  |  | X |  |  | X |  |
| 5 |  | X |  |  | X |  |  | X |  |  |
| 6 | X |  |  |  |  |  | X |  |  | X |
| 7 |  |  | X |  |  | X |  |  | X |  |
| 8 |  |  | X |  |  | X |  |  | X |  |
| 9 |  | X |  |  | X |  |  | X |  |  |
| 10 |  |  |  | X |  | X |  |  | X |  |
| 11 | X |  |  | X |  |  |  |  |  | X |
| 12 | X |  |  |  |  |  | X |  |  | X |
| 13 |  | X |  |  | X |  |  | X |  |  |
| 14 | X |  |  |  | X |  |  | X |  |  |
| 15 |  |  | X |  |  | X |  |  | X |  |
| 16 |  | X |  |  | X |  |  | X |  |  |
| 17 |  |  |  | X |  |  | X |  |  | X |
| 18 |  | X |  |  | X |  | X |  |  |  |
| 19 |  |  | X |  |  | X |  |  | X |  |
| 20 |  | X |  | X |  |  |  | X |  |  |

**Legend for table:** Min, minutes; h, hours.
